# Ceftolozane-tazobactam versus meropenem for definitive treatment of bloodstream infection due to extended-spectrum beta-lactamase (ESBL) and AmpC-producing Enterobacterales (“MERINO-3”): study protocol for a multicentre, open-label randomised non-inferiority trial

**DOI:** 10.1186/s13063-021-05206-8

**Published:** 2021-04-22

**Authors:** Adam G. Stewart, Patrick N. A. Harris, Mark D. Chatfield, Roberta Littleford, David L. Paterson

**Affiliations:** 1 Centre for Clinical Research, Faculty of Medicine, University of Queensland, Royal Brisbane and Women’s Hospital Campus, Brisbane, Queensland Australia; 2 Department of Infectious Diseases, Royal Brisbane and Women’s Hospital, Brisbane, Queensland Australia; 3 Department of Microbiology, Pathology Queensland, Royal Brisbane and Women’s Hospital, Brisbane, Queensland Australia

**Keywords:** Extended-spectrum beta-lactamase, AmpC beta-lactamase, Beta-lactam/beta-lactamase inhibitor, Carbapenem, Clinical trial

## Abstract

**Background:**

Extended-spectrum beta-lactamase (ESBL) and AmpC-producing Enterobacterales are common causes of bloodstream infection. ESBL-producing bacteria are typically resistant to third-generation cephalosporins and result in a sizeable economic and public health burden. AmpC-producing Enterobacterales may develop third-generation cephalosporin resistance through enzyme hyper-expression. In no observational study has the outcome of treatment of these infections been surpassed by carbapenems. Widespread use of carbapenems may drive the development of carbapenem-resistant Gram-negative bacilli.

**Methods:**

This study will use a multicentre, parallel group open-label non-inferiority trial design comparing ceftolozane-tazobactam and meropenem in adult patients with bloodstream infection caused by ESBL or AmpC-producing Enterobacterales. Trial recruitment will occur in up to 40 sites in six countries (Australia, Singapore, Italy, Spain, Saudi Arabia and Lebanon). The sample size is determined by a predefined quantity of ceftolozane-tazobactam to be supplied by Merck, Sharpe and Dohme (MSD). We anticipate that a trial with 600 patients contributing to the primary outcome analysis would have 80% power to declare non-inferiority with a 5% non-inferiority margin, assuming a 30-day mortality of 5% in both randomised groups. Once randomised, definitive treatment will be for a minimum of 5 days and a maximum of 14 days with the total duration determined by treating clinicians. Data describing demographic information, risk factors, concomitant antibiotics, illness scores, microbiology, multidrug-resistant organism screening, discharge and mortality will be collected.

**Discussion:**

Participants will have bloodstream infection due to third-generation cephalosporin non-susceptible *E. coli* and *Klebsiella* spp. or *Enterobacter* spp., *Citrobacter freundii*, *Morganella morganii*, *Providencia* spp. or *Serratia marcescens*. They will be randomised 1:1 to ceftolozane-tazobactam 3 g versus meropenem 1 g, both every 8 h. Secondary outcomes will be a comparison of 14-day all-cause mortality, clinical and microbiological success at day 5, functional bacteraemia score, microbiological relapse, new bloodstream infection, length of hospital stay, serious adverse events, *C. difficile* infection, multidrug-resistant organism colonisation. The estimated trial completion date is December 2024.

**Trial registration:**

The MERINO-3 trial is registered under the US National Institute of Health ClinicalTrials.gov register, reference number: NCT04238390. Registered on 23 January 2020.

**Supplementary Information:**

The online version contains supplementary material available at 10.1186/s13063-021-05206-8.

## Administrative information

The order of the items has been modified to group similar items (see http://www.equator-network.org/reporting-guidelines/spirit-2013-statement-defining-standard-protocol-items-for-clinical-trials/).

**Table:** 

Title {1}d	A Multicentre, Parallel Group Open-label Randomised Controlled Non-Inferiority Phase 3 Trial, of ceftolozane-tazobactam versus meropenem for definitive treatment of bloodstream infection due to Extended-Spectrum Beta-Lactamase (ESBL) and AmpC-producing Enterobacterales
Trial registration {2a and 2b}.	ClinicalTrials.gov identifier: NCT04238390
Protocol version {3}	Version 3, 17th of February 2020
Funding {4}	Merck Sharp & Dohme (Australia) Pty Limited investigator Initiated Funding.
Author details {5a}	Adam G Stewart^1,2^, Patrick NA Harris^1,3^, Mark D Chatfield^1^, Roberta Littleford^1^, David L Paterson^1,2^ ^1^Centre for Clinical Research, Faculty of Medicine, University of Queensland, Royal Brisbane and Women’s Hospital Campus, Brisbane, Queensland, Australia ^2^Department of Infectious Diseases, Royal Brisbane and Women’s Hospital, Brisbane, Queensland, Australia ^3^Department of Microbiology, Pathology Queensland, Royal Brisbane and Women’s Hospital, Brisbane, Queensland, Australia
Name and contact information for the trial sponsor {5b}	University of Queensland St Lucia Queensland Australia, 4072
Role of sponsor {5c}	The study sponsor is the University of Queensland. The Principal Investigator and the research team (authors) are responsible for the study design, collection, management, analysis, and interpretation of data and writing of the report or publication. The sponsor and the funder have no role in the study conduct, analysis and interpretation of the findings, and dissemination of the results.

## Introduction

### Background and rationale {6a}

Enterobacterales are common causes of bloodstream infection and may produce extended-spectrum beta-lactamases (ESBLs) or AmpC beta-lactamases. ESBL producers are typically resistant to third-generation cephalosporins such as ceftriaxone, but susceptible to carbapenems [1]. Among Enterobacterales, ESBLs have been found mainly in *Klebsiella* spp. and *Escherichia coli* but have also been reported in *Citrobacter* spp., *Enterobacter* spp., *Proteus* spp., *Providencia* spp., and *Serratia* spp. worldwide. Species with chromosomally encoded AmpC beta-lactamases may become resistant to third-generation cephalosporins due to hyper-expression of the enzyme following mutations in regulatory genes [2]. Common organisms harbouring chromosomal AmpC include *Enterobacter* spp., *Providencia* spp., *Serratia marcescens*, *Citrobacter freundii* and *Morganella morganii*. These organisms have also been found to co-harbour ESBLs. Plasmid-mediated AmpC beta-lactamases are less commonly encountered than ESBLS worldwide but can appear in bacteria isolated from patients after several days of hospitalisation [2]. Observational studies have been performed evaluating antibiotic choices for ESBL producers [3–10]. A landmark clinical trial comparing piperacillin-tazobactam to meropenem in the treatment of bloodstream infection due to ESBL-producing *E. coli* and *Klebsiella* spp. showed that piperacillin-tazobactam was not non-inferior to meropenem with respective to 30-day all-cause mortality [11]. A similar pilot clinical trial comparing these same antibiotics in the treatment of chromosomal AmpC-producers has finished recruitment [12]. In no observational study has the outcome of treatment for serious infections for ESBL or AmpC producers been significantly surpassed by carbapenems [3–10]. Despite the potential advantages of carbapenems for the treatment of ceftriaxone non-susceptible organisms, the widespread use of carbapenems may cause selection pressure leading to carbapenem-resistant organisms. This is a significant issue as carbapenem-resistant organisms are treated with last-line antibiotics such as colistin.

Ceftolozane-tazobactam is a combination of a new beta-lactam antibiotic with an existing beta-lactamase inhibitor, tazobactam, and is active against ESBL and most AmpC-producing organisms [13]. The most recent published data from the Study for Monitoring Antimicrobial Resistance Trends (SMART) global surveillance program in 2016 has revealed that 89.7% of Enterobacterales isolates were susceptible to ceftolozane-tazobactam, including 82.4% of ESBL-positive, carbapenemase-negative isolates [14]. In a large sample of ESBL- and AmpC-producing isolates from urinary tract and intra-abdominal specimens, over 80% of isolates tested susceptible to ceftolozane-tazobactam [15]. It has been FDA and EMA approved for complicated urinary tract infections (cUTI) and complicated intra-abdominal infections (cIAI), and more recently for hospital-acquired and ventilator-associated pneumonia (HAP/VAP) in 2019. In addition, a pooled analysis of phase 3 clinical trials has shown favourable clinical cure rates with ceftolozane-tazobactam for cUTI and cIAI caused by ESBL-producers [16]. Given the issues of carbapenem-resistant organisms, there is a need for establishing the efficacy of an alternative to carbapenems for serious infections.

### Objectives {7}

#### Primary objective

To compare the 30-day all-cause mortality of each treatment regimen; ceftolozane-tazobactam versus meropenem, in patients with bloodstream infection.

#### Secondary objectives

To compare each treatment regimen in relation to:

1. All-cause mortality at 14 days after randomisation
2. Clinical and microbiologic success (composite outcome)
3. Functional outcome
4. Rates of microbiological relapse
5. Growth of a new organism from blood cultures (not a contaminant) up to and including day 30
6. Length of hospital and ICU stay with each regimen
7. Treatment-emergent serious adverse events
8. Rates of *Clostridium difficile* infection (CDI) post treatment
9. Rates of colonisation and/or infection with multi-resistant bacterial organisms (MROs)
10. Desirability of Outcome Ranking (DOOR) with partial credit for both groups

### Trial design {8}

The study is an open-label randomised, controlled non-inferiority trial design comparing two drug regimens, meropenem versus ceftolozane-tazobactam randomised 1:1, for treatment of multidrug-resistant Gram-negative bloodstream infection.

## Methods: participants, interventions and outcomes

### Study setting {9}

The study is an international, multicentre hospital-based study, involving up to 40 sites and 630 participants randomised from Australia, Singapore, Spain, Italy, Lebanon, Saudi Arabia. A list of the study sites can be obtained from ClinicalTrials.gov (https://clinicaltrials.gov/ct2/show/NCT04238390?cond=ceftolozane+meropenem&draw=2&rank=1).

### Eligibility criteria {10}

#### Inclusion criteria

1. Qualifying bloodstream infection is defined as at least one peripheral blood culture draw demonstrating Enterobacterales with proven non-susceptibility to third-generation cephalosporins or a cephalosporin susceptible chromosomal AmpC-producing Enterobacterales (*Enterobacter* spp., *Klebsiella aerogenes*, *Citrobacter freundii*, *Morganella morganii*, *Providencia* spp. or *Serratia marcescens*). Non-susceptibility to third-generation cephalosporins (any one of ceftriaxone, cefotaxime or ceftazidime) is defined by susceptibility breakpoints used in the local testing laboratory (either EUCAST or CLSI). Bacterial identification to species level will be performed using standard laboratory methods (e.g. MALDI-TOF) and susceptibility testing (e.g. VITEK2) according to local practice and standards. This may include newly validated methods such as the Accelerate Pheno™ system
2. Participant is aged 18 years and over (21 and over in Singapore)
3. The participant or approved proxy is able to provide informed consent
4. ≤ 72 h has elapsed since the first positive qualifying (index) blood culture collection
5. Expected to receive IV therapy for ≥ 5 days

#### Exclusion criteria

1. Known hypersensitivity to a cephalosporin or a carbapenem, or anaphylaxis to beta-lactam antibiotics
2. Participant with significant polymicrobial bloodstream infection (i.e. not a contaminant)
3. Treatment is not with the intent to cure the infection (i.e. palliative intent) or the expected survival is ≤ 5 days
4. Participant is pregnant or breast-feeding
5. Use of concomitant antimicrobials with known activity against Gram-negative bacilli (except trimethoprim/sulfamethoxazole for Pneumocystis prophylaxis and when adding metronidazole for suspected IAI) in the first 5 days post-randomisation
6. Participants with creatinine clearance (CrCl) < 15 ml/min (determined by Cockcroft-Gault formula or Modification of Diet in Renal Disease (MDRD) formula) or on renal replacement therapy
7. Previously randomised in the MERINO-3 trial or concurrently enrolled in another therapeutic antibiotic clinical trial
8. Blood culture isolate with in vitro resistance to either meropenem or ceftolozane-tazobactam (known either at time of enrolment or during the course of study treatment, in which case the participant will be withdrawn)

### Who will take informed consent? {26a}

Potential participants will be identified by treating clinical teams in collaboration with investigating research teams. Once the investigating team has been notified of a potential eligible participant (as determined by the microbiology laboratory) by the treating team, a member of the research team will visit the participant at the bedside. The initial screening visit will include a clinical record review, discussion with the treating team and brief participant interview to determine suitability for inclusion. The Principal Investigator/delegate will discuss the risks, benefits and procedures of the trial. The participant will be given an opportunity to ask questions regarding the study and will receive a copy of the HREC approved and updated consent form with his/her signature. The right of a participant to refuse participation without giving reasons will be respected.

Alternative methods for supporting the informed consent process will be employed in the event of inability to read and write, require translation or have cognitive impairment. Approved substitute decision-maker (SDM) consent processes will be provided. The participant/SDM are free to withdraw from the trial at any time without giving reasons and without prejudicing the participant’s further treatment.

All written material and hospital translational services will be provided in an appropriate language for the participant/SDM. The Investigator retains overall responsibility for the conduct of research at their site, this includes the taking of informed consent of participants at their site. They must ensure that any person delegated responsibility to participate in the informed consent process is duly authorised, trained and competent to participate according to the ethically approved protocol, principles of Good Clinical Practice (GCP) and Declaration of Helsinki.

### Additional consent provisions for collection and use of participant data and biological specimens {26b}

The investigators do not expect to conduct ancillary studies requiring the use of participant data that is collected in this study.

### Interventions

#### Explanation for the choice of comparators {6b}

Carbapenems are standard of care for treatment of ESBL and AmpC-producing Enterobacterales infection. Observational studies have been performed evaluating antibiotic choices for ESBL producers [3–10]. The MERINO trial failed to demonstrate non-inferiority of piperacillin-tazobactam 4.5 g every 6 h, when compared to meropenem 1 g every 8 h in bloodstream infection due to ceftriaxone non-susceptible *E. coli* and *Klebsiella* species [11].

#### Intervention description {11a}

Ceftolozane–tazobactam will be packaged, labelled, QP released and distributed by Merck, Sharpe and Dohme (MSD) in accordance with regulations and Good Manufacturing Practice. Ceftolozane-tazobactam will be distributed and stored using cold chain logistics.

Meropenem will be obtained from local hospital pharmacy inventory and will be prescribed and administered within its marketing authorisation (MA) will be labelled in accordance with the requirements for a dispensed medicine.

Meropenem 1 g or ceftolozane-tazobactam 3 g, will both be administered every 8 h intravenously as per product information guidelines. Meropenem will be administered over 30 min and ceftolozane-tazobactam over 60 min.

#### Criteria for discontinuing or modifying allocated interventions {11b}

Duration of study drug administration will be at the discretion of the treating clinician and consent of the participant for a minimum of 5 days to a maximum of 14 days. Dose adjustment for renal impairment will be made according to the criteria below. Patients receiving renal replacement therapy or those with a creatinine clearance < 15 ml/min are excluded from the trial. Blinding will not be performed.

Participants that have received at least one dose of allocated study medication and have primary outcome data will be considered evaluable.

##### Renal function-guided treatment dose and frequency

**Table:** 

Creatinine clearance (mL/min)	Ceftolozane-tazobactam	Meropenem
> 50	3 g every 8 h	1 g every 8 h
30–50	1.5 g every 8 h	1 g every 12 h
15–29	750 mg every 8 h	500 mg every 12 h

#### Strategies to improve adherence to interventions {11c}

Participants will be in-patients for intervention; therefore, control of drug dose will be by the bedside clinical team and participants consent.

#### Relevant concomitant care permitted or prohibited during the trial {11d}

Other antimicrobials active against Gram-negative bacilli are excluded in the first 5 days after enrolment, except trimethoprim-sulfamethoxazole which may be continued as *Pneumocystis* prophylaxis. Addition of metronidazole for suspected intra-abdominal infection is permitted. Ongoing treatment of prior Gram-positive infections will be limited to agents without concomitant Gram-negative activity.

#### Provisions for post-trial care {30}

Randomised participants can receive a minimum of 5 days to a maximum of 14 days of allocated treatment. Participants may meet the criteria of withdrawal any point post randomisation during the treatment period. On experiencing an adverse event, completion, or early withdrawal of study treatment the treating clinician will direct any required routine clinical care.

#### Outcomes {12}

The following tables describe the primary and secondary outcomes and criteria of evaluation (Tables 1 and 2).

**Table 1:** Primary objective and outcome

Primary objective and outcome measure		
Objective	Outcome measure	Time point(s) of evaluation
To compare 30-day all-cause mortality of 5 to 14 days ceftolozane-tazobactam versus meropenem for definitive treatment of bloodstream infection due to ESBL or AmpC-producing Enterobacterales	Difference in proportion of those dead in each group	30 days after randomisation

**Table 2:** Secondary objectives and outcomes

Secondary objectives and outcome measures			
#	Objectives	Outcome measures	Time point(s) of evaluation
1	To compare 14-day all-cause mortality of each regimen	Difference in proportion of those dead in each group	Day 14
2	To compare clinical and microbiologic success of each regimen at day 5	Difference in proportion of those achieving clinical and microbiological success in each group; defined as: 1. Participant alive 2. Fever resolved (< 38 °C) 3. SOFA score (ICU) or modified SOFA score (non-ICU) improved 4. Absence of growth of index organism	1. Day 5 2. Day 5 3. Day 1 and day 5 4. Up to and including day 5
3	To compare the functional outcome of patients treated with each regimen	Difference in mean change between baseline and 30-day post-randomisation FBS between groups NB. Baseline reflects pre-admission status prior to condition meriting hospital admission.	Screening and day 30
4	To compare the rates of relapse of bloodstream infection (microbiological failure) with each regimen	Difference in proportion who experience growth of the same organism as index blood culture between groups	Up to Day 30
5	To compare the rates of new bloodstream infection with each regimen	Difference in the proportion who experience growth of a new organism from blood cultures (not a contaminant) between groups	Up to Day 30
6	To compare lengths of inparticipant hospital (acute) and ICU stay with each regimen [not including inparticipant rehabilitation, long term acute or Hospital in the Home (HITH)]	Difference in median ICU ± non-ICU length of hospital stay between groups	Cumulative up to day 30
7	To compare the number of treatment-emergent serious adverse events with each regimen	Difference in proportion of treatment-emergent serious adverse events between groups	Day 1 to the last dose plus 24 h
8	To compare rates of CDI with each regimen	Difference in proportion of clinician diagnosed (including a positive CDI test) and treated CDI between groups	30 days
9	To compare rates of colonisation and/or infection with multi-resistant bacterial organisms (MROs) including those newly acquired	Difference in proportion of those with MROs identified between groups; defined as: 1. Known previous or current colonisation and/or infection with MRO. 2. MROs detected from any clinical specimen. MROs include vancomycin-resistant Enterococci (VRE), methicillin-resistant *Staphylococcus aureus* (MRSA) and multi-resistant gram negative organisms including carbapenem-resistant Enterobacterales (CRE), carbapenemase-producing Enterobacterales (CPE), carbapenem-resistant *Pseudomonas aeruginosa* (CRP), carbapenem-resistant Acinetobacter baumannii (CRAB)	Baseline and up to day 30
10	To compare the Desirability of Outcome Ranking (DOOR) with partial credit with each regimen	Mean difference in DOOR between groups at Day 30 post-randomisation. This is based on: 1.Vital status (alive or dead) 2. *C. difficile* infection 3. CRE colonisation 4. Functional status (i.e. FBS)	Day 30

N.B. A priori, all endpoints will be assessed both for the entire study population and for the study population with bloodstream infection due to (1) *E.coli* and *Klebsiellae* spp. or (2) chromosomal AmpC-producer (*Enterobacter* spp., *Klebsiella aerogenes*, *Citrobacter freundii*, *Morganella morganii*, *Providencia* spp. or *Serratia marcescens*)

### Participant timeline {13}

The participant timeline is shown in Table 3.

**Table 3:**
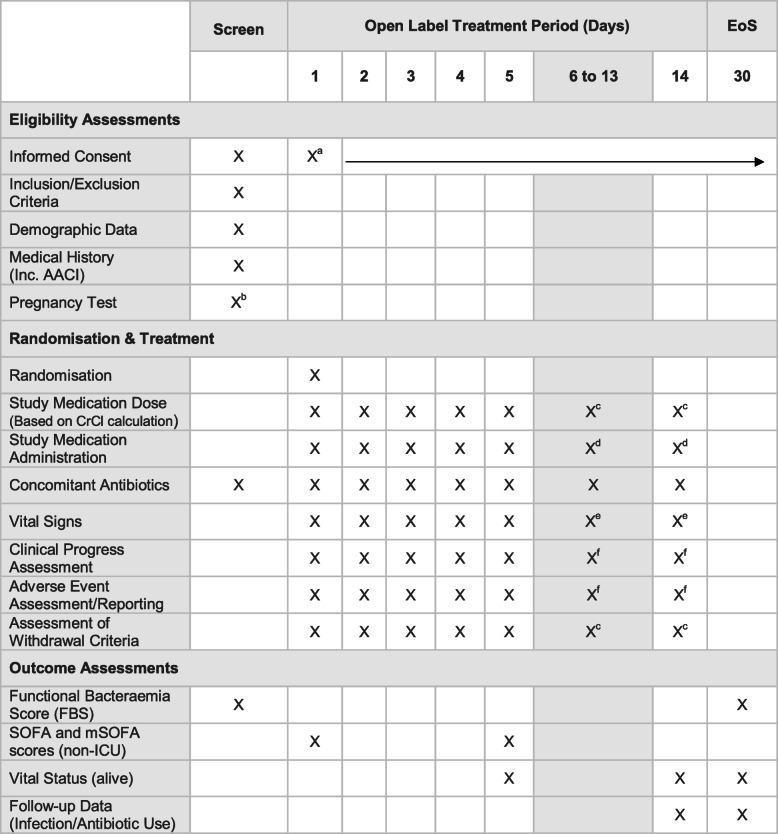
Study time and event schedule

^a^Written informed consent at screening or day 1 prior to any study procedure. Continue to obtain verbal consent for the continuation of participation

^b^ Females of child-bearing potential. Only required if routine clinical pregnancy test result is not available. Urine or blood test as per local practice. Where possible in unison with other routine clinical tests

^c^If participant remains/remained on study treatment

^d^Clinician decision to cease or continue study medication up to and including day 14

^e^Only if the participant remains in the hospital

^f^ If participant remains on study treatment or within 24 h of the last dose of study treatment

NB. Index blood culture and any subsequent blood cultures will be taken as part of routine clinical practice during the 30-day trial period if the participant is febrile—defined as temperature ≥ 38.0 °C

Routine clinical haematology and biochemistry results will be used to calculate SOFA, mSOFA and CrCl

### Sample size {14}

The sample size is driven by the maximum achievable power with the constraint of a predefined quantity of ceftolozane-tazobactam to be supplied by MSD. We anticipate that a trial with 600 patients contributing to the primary outcome analysis (corresponding to 630 patients randomised) may be possible. Such a trial would have 80% power to declare non-inferiority with a 5% non-inferiority margin, assuming a 30-day mortality of 5% in both randomised groups. The decision to choose 5% as the expected 30-day mortality reflects the 30-day mortality rate of 4–5% reported by our group in the meropenem arm of the MERINO trial [11]. However, a similar study has assumed a 30-day mortality rate of 12.5% in the meropenem arm [17]. This mortality rate was based on both arms of the MERINO trial and contemporary observational studies [4]. If the control rate is higher than 5% we will have less power to detect non-inferiority (assuming equivalent rates in the two arms). If the control rate is 5%, the non-inferiority margin of 5% may be deemed by some to be too large. As recommended by the CONSORT statement, we will report relative risk ratios as well as absolute risk differences.

#### Recruitment {15}

We plan to conduct this trial over a 4-year period. Participants will be recruited from a maximum of 40 sites across 6 countries. Of those participants screened, we anticipate approximately 25% will be enrolled successfully in the trial. This estimate was generated from the MERINO trial.

The table below highlights the minimum projected monthly and annual recruitment numbers to reach target (630) depending upon the number of sites. It assumes all sites are recruiting simultaneously, however, it is projected that the majority of the sites will be recruiting within a 9-month period, and the early adoptive sites will recruit ahead of the initial target thereby balancing timelines.

In the event of delayed recruitment milestones, pre-defined contingency measures including, the addition of eligible sites and the introduction of further rapid antibiotic susceptibility testing (e.g. AcceleratePheno™) will be implemented (Table 4).

**Table 4:** Predicted recruitment per site and timeline

# Sites	Per month	Per annum
1	13.1	157.5
10	1.3	15.7
15	0.9	10.5
20	0.7	7.9
25	0.5	6.3
30	0.4	5.2
35	0.4	4.5
40	0.3	3.9

### Assignment of interventions: allocation

#### Sequence generation {16a}

A computer-generated random sequence will be generated using random permuted blocks of unequal length. Participants will be stratified according to infecting infectious syndrome (urinary tract vs other) and economic region (high vs middle income according to Organisation for Economic Cooperation and Development (OECD) definitions (http://www.oecd.org/dac). The allocation sequence will be stored in REDCap and concealed from all personnel involved in the trial and will be generated by the trial statistician who is not involved in clinical care.

#### Concealment mechanism {16b}

The randomisation list will be generated centrally by an independent statistician, kept confidential, and imported through the REDcap trial database.

#### Implementation {16c}

Eligible participants will be randomly assigned to either ceftolozane-tazobactam or meropenem in a 1:1 ratio according to a randomisation list prepared in advance by the trial statistician. Participants will be randomised to either meropenem or ceftolozane-tazobactam in a 1:1 ratio. According to a randomisation list prepared in advance. The randomisation process will be managed by The Clinical Trial Coordinating Centre (UQCCR) via an online module within the REDCap data management system. Once randomised, the first dose of the study drug will be administered by clinical ward staff.

### Assignment of interventions: blinding

#### Who will be blinded {17a}

This will be an open-label trial, with the participant, investigator, site study and project management teams being aware of treatment allocation. Issues that were considered justification for an open-label design included the study’s endpoint (mortality) which is considered a hard endpoint that is not subjective thereby limiting the risk associated with the need to adjust blinded drugs with different pharmacokinetics and dynamics in patients with renal dysfunction. Overall, the open-label trial will provide a population and intervention with greater generalisability, and not compromise internal or external validity.

An independent statistician will create the randomisation list. Permissions will be set in REDcap so that the statistician will not see the list or treatment allocations. Trial investigators will be protected from seeing the data analysis split by group. An independent statistician will make a confidential report for the DSMB.

#### Procedure for unblinding if needed {17b}

There will be no unblinding procedures undertaken.

### Data collection and management

#### Plans for assessment and collection of outcomes {18a}

Demographic, clinical and laboratory data will be entered into the study electronic case report form (eCRF). Study visits will continue on days 2–14 and will involve collecting clinical and laboratory data to be entered into the study eCRF. Daily recording of clinical parameters, concomitant antibiotic therapy and adverse events will continue until and including day 14 post-randomisation or until cessation of study treatment. On day 30, all primary and secondary outcomes will be determined. This will primarily involve a review of all clinical and laboratory records for that period. It may involve a telephone consultation if the participant has been discharged. There will be no requirement for additional hospital visits or tests. The study team will utilise clinical data and participant reporting to calculate validated scoring systems including; Sequential Organ Failure Assessment (SOFA), or modified SOFA at day 1 and day 5. The Functional Bacteria Score (FBOS) will be calculated using pre-admission and day 430 physical status (Tables 5, 6, 7, 8 and 9).

**Table 5:** Functional Bacteremia Score (FBS) [18]

Function	Score
Out of hospital; basically healthy; able to work or perform usual activities	7
Out of hospital; moderate signs or symptoms of disease; unable to work or perform usual activities	6
Out of hospital; significant disability; requires a high level of care and assistance daily	5
Hospitalised but not requiring ICU	4
Hospitalised in ICU	3
Accommodated in a long-term ventilator unit	2
On palliative care in terminal phases of life (in hospital or at home)	1
Dead	0

**Table 6:** Exploratory “DOOR” endpoint with partial credit [19]

Composite	Score
Dead within 14 days of randomisation	0
Dead within 30 days of randomisation	10
Dead within 30 days, and with *C. difficile* or carbapenem-resistant Enterobacterales (CRE) during the time from randomisation to death	20
Dead within 30 days, but without *C. difficile* or CRE during the time from randomisation to death	30
Alive at 30 days, but with deterioration in functional status, and with *C. difficile* or CRE	50–65*
Alive at 30 days, but with deterioration in functional status, but no *C. difficile* or CRE	70–85*
Alive at 30 days, return to baseline functional status but with *C. difficile* or CRE	90
Alive at 30 days, return to baseline functional status and no *C. difficile* or CRE	100

*5 point penalty for each decrement in functional bacteremia score (e.g. 85 = 1 FBS score decrement in a participant without *C. difficile* or CRE, 80 = decrement of 2 on FBS, 75 = decrement of 3 on FBS, 70 = decrement of 4 or more on FBS)

**Table 7:** Sequential Organ Failure Assessment (SOFA) Score [20]

Organ/system	0	1	2	3	4
SpO2/FiO2	> 400	< 400	< 300	< 200 and mechanical ventilation	< 100 and mechanical ventilation
Bilirubin (μmol/L)	< 20	20–32	33–101	102–204	> 204
Hypotension	MAP ≥ 70 mmHg	MAP < 70 mmHg	Dopamine ≤ 5 μ/kg/min or dobutamine any dose	Dopamine > 5, adrenaline ≤ 0.1, noradrenaline ≤ 0.1	Dopamine > 15, adrenaline > 0.1, noradrenaline > 0.1
Platelets (× 10^9^)	≥150	< 150	< 100	< 50	< 20
GCS	15	13–14	10–12	6–9	< 6
Creatinine (mg/dL)	< 1.2	1.2–1.9	2.0–3.4	3.5–4.9	> 5.0

**Table 8:** Modified Sequential Organ Failure Assessment (mSOFA) Score [21]

Organ/system	0	1	2	3	4
SpO2/FiO2	> 400	≤ 400	≤ 315	≤ 235	≤ 150
Liver	No scleral icterus			Scleral icterus or jaundice	
Hypotension	No hypotension	MAP < 70 mmHg	Dopamine ≤ 5 or dobutamine any dose	Dopamine > 5, adrenaline ≤ 0.1, noradrenaline ≤ 0.1	Dopamine > 15, adrenaline > 0.1, noradrenaline > 0.1
GCS	15	13–14	10–12	6–9	< 6
Creatinine (mg/dL)	< 1.2	1.2–1.9	2.0–3.4	3.5–4.9	> 5.0

**Table 9:** Age-Adjusted Charlson Comorbidity Index (ACCI) [22]

Age	< 50	0
	50–59	+ 1
	60–69	+ 2
	70–79	+ 3
	≥ 80	+ 4
Myocardial infarction	No 0	Yes + 1
Chronic heart failure	No 0	Yes + 1
Peripheral vascular disease	No 0	Yes + 1
Cerebral vascular accident or transient ischemic attack	No 0	Yes + 1
Hemiplegia	No 0	Yes + 2
Dementia = (chronic cognitive impairment)	No 0	Yes + 1
Chronic obstructive pulmonary disease	No 0	Yes + 1
Connective tissue disease	No 0	Yes + 1
Peptic ulcer disease	No 0	Yes + 1
Liver disease	None	0
	Mild	+ 1
	Moderate to severe	+ 3
Diabetes mellitus	None or diet controlled 0	
	Uncomplicated	+ 1
	End-organ damage	+ 2
Moderate to severe chronic kidney disease	No 0	Yes + 1
Solid tumour	None	0
	Localised	+ 2
	Metastatic	+ 6
Leukaemia	No 0	Yes + 2
Lymphoma	No 0	Yes + 2
AIDS	No 0	Yes + 2

#### Plans to promote participant retention and complete follow-up {18b}

Participants or if appropriate their representative (SDM), are free to prematurely discontinue treatment or withdraw from the study at any point, or a participant can be withdrawn by the investigator or at the request of the treating physician or by meeting a specified withdrawal exclusion criterion.

The defined premature treatment discontinuation criteria include:

(i) CrCl < 15 mL/min or commenced on renal replacement therapy

(ii) During the course of study treatment - blood culture isolate with in vitro resistance to allocated treatment

If withdrawal occurs, the primary reason for withdrawal will be documented, if possible, in the participant’s case record form. The participant will have the option to withdrawal from:

(i) Study medication with continued data collection

(ii) All aspects of the trial but continued use of data collected up to that point.

If premature treatment discontinuation occurs as a result of a treatment-emergent adverse event, appropriate medical care will continue to be provided by the primary treating team.

Participants who are withdrawn or who have their treatment discontinued prematurely during intervention and participants who do not provide consent to remain in the trial will not be replaced.

However, rates of withdrawal and premature treatment discontinuation will be monitored. If withdrawal or premature treatment discontinuation rates are higher than anticipated, a strategy may be required to ensure study power is maintained. If applicable, any protocol amendment will be submitted for regulatory approval.

Participants/SSDM who revoke consent will be assured that such action will not jeopardise their care or their relationship with their treating team.

Participants that have received at least one dose of allocated study medication and have primary outcome date will be considered evaluable.

#### Data management {19}

UQCCR will be responsible for data management and quality. A data management plan will be prepared to cover data entry, coding, security and storage, including quality control.

A clinical database using the REDCap an Electronic Data Capture (EDC) web-based system has been developed with a web hosting facility. Electronic case report forms (eCRFs) have been developed and validated to collect all clinical and laboratory-related information. The trial database will include information on demographics (age, gender), underlying illnesses, baseline and follow-up laboratory data including microbiologic data (e.g., organism type, mechanism of resistance and minimal inhibitory concentration (MIC) of study drug), and daily assessments including; vital signs, blood results and adverse events and treatments for the purpose of assessment of clinical and primary outcome.

Data for this study will be recorded using REDCap. Data will be stored in a re-identifiable manner in the database using a unique screening number for each participant. The database will contain validation ranges for each variable to minimise the chance of data entry errors. An audit trail will maintain a record of initial entries and changes made; reasons for change; time and date of entry; and user name of person who made the change. Data queries will be raised by the project manager/delegate and missing data or suspected errors will be raised as data queries and resolved prior to database lock and analysis. The database will contain in-line capability so that these queries and answers are logged as part of the audit trail. Individuals will be trained and issued log-in details and access will be restricted to necessary fields only. The study teams at site and individuals at UQCCR involved in follow-up data collection will enter data.

Paper data collection sheets generated from REDCap may be used by site staff if required, ensuring that all data is entered onto the eCRF in a timely manner.

Following each study visit, the designated site staff will complete the visit specific eCRF. Once all required information is received the eCRF shall be considered complete. Trial Management staff will then monitor the data for completeness and accuracy. Any eCRF discrepancies, either manual or automatic, will be addressed with the site staff for clarification.

REDCap is held on a specific server at the University of Queensland using standard industry SSL to ensure data privacy as per UQ UQ Cyber Security Policy and Procedures. The database is back-up daily to a secure file server. Governance of the data is restricted to project staff and REDCap server administrators as required by the project and functioning of the REDCap system, including server maintenance, security and backup.

Any electronic data records stored locally will be kept only on a single computer located within the relevant department, using a password-protected folder. The PI will keep any paper-based records, study files or source documentation in a locked cabinet within the department. These records, electronic and physical, will be kept for a minimum of 15 years after the completion of the trial before being destroyed or erased, as per NHMRC guidelines. These documents will be retained for a longer period if required by the applicable regulatory requirements or institutional policy.

#### Confidentiality {27}

The following personal data will be collected at the site as part of the research:

Participant’s name, address, phone number, date of birth will be collected. If appropriate, the name, address and phone number of the person acting as the legal representative will also be collected.

Personal data will be stored securely by the research team at each recruiting site for up to 10 years after the study has finished. Where this information includes identifiable information, it will be held securely with strict access arrangements as per local confidentiality laws and regulations.

No personal data will be transferred to UQCCR, except for the date of birth in the event of safety reporting processing of personal data for the purposes of pharmacovigilance is necessary determined as the legal basis for public interest under the EU General Data Protection Regulations (GDPR). All data will be held securely with strict access arrangements. The participant will be informed of study-related data that will be collected, stored, transferred and used in accordance with local and appropriate national data protection law. The level of disclosure will be explained to the participant who will be required to give consent for their data to be used as described.

Data will be collected on study-specific electronic and paper copy case record forms. The Investigator is responsible for ensuring the data collected are complete, accurate and recorded in a timely manner.

Source documents are those where data are first recorded, and from which participants’ case report form (CRF) data are obtained. These include but are not limited to hospital records both electronic and paper, which will include medical history, previous and current medications, and any relevant laboratory test results, participant progress notes, pharmacy records and any other reports or records of procedures performed in accordance with the protocol. A further potential data source will be through telephone conversations with the study participant or SDM.

Any document that acts as a source document (the point of the initial recording of a piece of data) should be signed and dated by the person recording or reviewing the data for issues of medical significance (for example the review of laboratory reports). Persons signing the source documents must be listed as a site staff member.

The sponsor’s monitor/designee will either visit sites or conduct remote source document verification. The number of visits will be outlined in the study monitoring plan.

### Plans for collection, laboratory evaluation and storage of biological specimens for genetic or molecular analysis in this trial/future use {33}

Blood cultures and other blood laboratory tests will be collected as per local clinical procedures, using standard blood culture bottles and recommended blood volumes, an EDTA tube (4–6 ml) for FBC, and a lithium heparin tube (4–6 ml) for LFTs, EUC, CRP. The clinical team will arrange these tests as part of usual care but the research team will ensure blood is collected and analysed. A mandatory FBC and EUC will be requested on days 1 and 5 post-randomisation by the study investigators, if not already obtained by the treating team. No specific blood or urine tests are required in addition to standard clinical care (with the exception of a pregnancy test, if required). The study will collect routine laboratory data and enter it into the eCRF.

All blood cultures which flag positive will be processed as per the local laboratory’s usual procedures. Microbiology laboratories at study sites will perform susceptibility testing for ceftolozane-tazobactam for each entry blood culture isolate. This may be done by any validated method that is consistent with clinical practice at the site. This may be in addition to standard laboratory procedures for antibiotic susceptibility testing in some laboratories. Trial investigators will be able to assist laboratories in performing ceftolozane-tazobactam susceptibility testing by (1) supplying new VITEK2® automated susceptibility testing cards with ceftolozane-tazobactam inbuilt and (2) asking the labs to set up a ceftolozane-tazobactam disk diffusion or gradient (e.g. Etest) susceptibility tests which will be provided.

All bacterial isolates will be frozen and stored as per standard laboratory practice at each site. Most laboratories store all sterile site isolates routinely, but to ensure the availability of relevant isolates for study procedures, the microbiology laboratory at each site will be asked to freeze and store as per MERINO-3 Manual of Operations. Isolates from the index blood culture and any subsequent cultures obtained during the first 30 days after randomisation will be collected. These will later be transported for testing and genomic analysis Isolates will be transported to UQCCR in batches and specified time points in the study. Isolates will be identified by their MERINO-3 Participant ID and local laboratory specimen ID number. They will be transported either as colonies subcultured onto agar slopes or as cotton swabs which have picked up an individual colony of a pure subculture of the organism. It is the responsibility of each site’s Principal Investigator to ensure that all site staff handling, packaging, and/or shipping biological samples understand and comply with International Air Transport Association (IATA) regulations relating to the handling and shipping of hazardous goods and/or diagnostic specimens.

### Statistical methods

#### Statistical methods for primary and secondary outcomes {20a}

Thirty-day all-cause mortality will be reported as *n*/*N* (%) for each randomised group. The absolute difference in these percentages will be reported, along with a two-sided 95% CI. If this 95% CI excludes the inference at the population level that 30-day all-cause mortality is ≥ 5% higher for patients receiving ceftolozane-tazobactam compared to meropenem, then we will declare non-inferiority of ceftolozane-tazobactam with a 5% non-inferiority margin. In such a situation, we will also declare superiority of ceftolozane-tazobactam if the 95% CI excludes 30-day all-cause mortality being ≥ 0% higher for patients receiving ceftolozane/tazobactam.

Plans for the statistical analysis and reporting of each secondary outcome will be decided upon by investigators with access to pooled data.

#### Interim analyses {21b}

An interim analysis—including both efficacy and safety endpoints—will be performed after the first 25 then 50 subjects have completed the 30-day study period. The timing of additional interim analyses will be determined by the DSMB. The Committee will not have executive power to stop the trial or modify treatment but can make a recommendation for the former or latter. The DSMB will provide reports of its observations and recommendations to the TMG and lead HREC Committee.

#### Methods for additional analyses (e.g. subgroup analyses) {20b}

The primary outcome analysis will be undertaken in the following sub-groups:

1. urinary versus non-urinary source (bloodstream infections due to a urinary source are usually less severe and associated with more favourable mortality outcomes; it has been hypothesised that there is less of a treatment effect difference between beta-lactam/beta-lactamase inhibitors and carbapenems in this group)
2. Pitt bacteremia score ≥ 4 versus < 4 (Pitt bacteremia score is used as a severity acute illness index; it has been hypothesised that patients with less severe acute illness demonstrate less of a treatment effect difference between beta-lactam/beta-lactamase inhibitors and carbapenems)
3. Appropriate versus inappropriate empirical therapy (defined as in vitro activity against target organism) (those receiving appropriate empirical therapy have improved clinical outcomes; it has been hypothesised that there a diminished treatment effect difference between beta-lactam/beta-lactamase inhibitors and carbapenems in this group)
4. OECD country income (middle versus high) (Those receiving medical care in a high-income country may have improved clinical outcomes related to more accurate and early antibiotic susceptibility results which may explain diminished treatment effect difference between beta-lactam/beta-lactamase inhibitors and carbapenems in this group)
5. *E. coli* and *Klebsiella* spp. versus chromosomal AmpC-producers (Ceftolozane-tazobactam has better in vitro activity overall against ESBL producing Enterobacterales when compared with AmpC-producing bacteria; a diminished treatment effect difference may be seen in those with *E. coli* and *Klebsiella* spp. bloodstream infection)
6. Immune compromise versus non-immune compromise (Improved clinical outcomes and reduced acute severity of illness is seen in non-immune compromise patient; there may be a diminished treatment effect difference between beta-lactam/beta-lactamase inhibitors and carbapenems in this group)
7. Healthcare-associated infection versus non-healthcare associated infection (healthcare-associated infections are often caused by bacteria with multiple different mechanisms of resistance, in addition to being more unwell when compared to non-healthcare associated; a greater difference in treatment effect may be observed between beta-lactam/beta-lactamase inhibitors and carbapenems in this group)

Heterogeneity of treatment effect (on the odds ratio scale) will be explored across sub-groups using a test for the intervention × subgroup interaction by adding this term and the subgroup as covariates in a logistic regression model.

There is no intention to perform or consider adjusted analyses.

#### Methods in analysis to handle protocol non-adherence and any statistical methods to handle missing data {20c}

Study participants who receive at least one dose of study drug will be included in the modified intention-to-treat (mITT) population. Participants who receive at least 5 days of study drug will be included in the per-protocol (PP) population. Patients who are randomised but do not receive study drug will be excluded. The primary outcome will be determined for the mITT and PP populations. We expect missing data to be minimal for all outcomes, however, if primary outcome data is missing it will not be included in the mITT analysis.

#### Plans to give access to the full protocol, participant level-data and statistical code {31c}

The detailed statistical analysis methods will be specified in a Statistical Analysis Plan (SAP) and will be submitted as supplementary material along with the final manuscript. For the analyses changed from those outlined in the protocol, the reason for changes from the protocol will be described in the SAP. The first draft of the SAP will be available before the first participant is dosed, and any subsequent minor changes to the SAP will be finalised before database lock.

### Oversight and monitoring

#### Composition of the coordinating Centre and trial steering committee {5d}

The University of Queensland will act as the study Sponsor taking overall responsibility for the conduct of the trial. The Coordinating Principal Investigator, with the coordinating centre, UQCCR will undertake sponsor delegated duties and functions for study design, data collection, trial management, safety reporting, analysis and interpretation, manuscript and clinical report writing, and dissemination of results.

Separate agreements will be put in place between the sponsor and each organisation undertaking duties and functions in relation to participation in the study.

As the coordinating centre, UQCCR will establish a Trial Management Group (TMG), chaired by Coordinating Principal Investigator/Delegate. It will include representatives from the coordinating centre, coinvestigators, and a consumer/participant representative. The day-to-day operational activities will be managed by the UQCCR Co-Investigators and Project Manager/Delegate and are accountable to the Coordinating Principal Investigator.

The TMG roles and responsibilities will be detailed Charter.

Investigators and institutions involved in the study will permit trial-related monitoring and audits on behalf of the sponsor, IRB/REC and regulatory inspection(s). In the event of an audit or monitoring, the Investigator agrees to allow the representatives of the sponsor direct access to all study records and source documentation. In the event of regulatory inspection, the Investigator agrees to allow inspectors direct access to all study records and source documentation.

A study-specific risk assessment will be performed by the Trial Management Team. Input will be sought from the CPI or designee. The outcomes of the risk assessment will form the basis of the monitoring plans and audit plans. The risk assessment will be reviewed in light of protocol amendments, breaches and any other significant information that could impact trial risk.

In principle the project will be monitored to varying degrees by the CPI, investigators, Coordinators, Project Manager/delegate, TMG, DSMC and HREC.

Specific monitoring will be conducted by clinical trial monitors, or designees, who will perform monitoring activities in accordance with the study monitoring plan. This will involve on-site visits and remote monitoring activities as necessary including; site file review, review of informed consent forms, source data verification (SDV) and serious adverse event (SAE) review as per monitoring plan objectives. Investigator site audits, study management audits and facility (including 3rd parties) audits will be conducted by the relevant trained individuals as required including; sponsor/designee, regulators, IRBHREC representatives as necessary.

#### Composition of the data monitoring committee (DMC), its role and reporting structure {21a}

This trial will be monitored by a DSMB. The DSMB, but not investigators, will be able to see summaries of accumulating data split by randomised group. Interim analyses will be supplied, in strict confidence, to the DSMB, as per the DSMB Charter and as per Chair request. The DSMB is independent of the trial organisers. The Charter for the DSMB will be agreed at their first meeting. Meetings of the committee will be arranged periodically, as considered appropriate by the Chair. In light of interim data on the trial’s outcomes, adverse event data, accumulating evidence from other trials, the DSMB will inform the Trial Management Group (TMG) if in their view there is proof beyond reasonable doubt that the data indicate that any part of the protocol under investigation is either clearly indicated or contra-indicated, either for all adult participants or for a particular subgroup of trial participants. Unless modification or cessation of the trial is recommended by the DSMB, the TMG, collaborators and administrative staff (except those who supply the confidential information) will remain uninformed of the results of the interim analysis. Collaborators and all others associated with the study may write to the DSMB to draw attention to any concern they may have about the possibility of harm arising from the treatment under study. A guideline for the DSMB recommending early termination of the trial on the basis of harm, at any interim analysis, is a worse primary outcome for patients receiving ceftolozane-tazobactam with *p* < 0.01 (i.e. < 1/100) (Fisher’s exact test).

The interim analysis will review outcome data and answer the following questions:

1. Are there any significant safety issues that may present an ethical issue in continuing the study? This may include adverse events, but also study conduct and protocol breaches.

2. Are there overwhelming data suggesting the superiority of one arm that may present an ethical issue in continuing the study?

3. Are there any other factors that may impact on the feasibility/usefulness of the study? For example, rate of enrolment, unexpected low rate of outcomes, unable to fund, protocol violations, etc.

Any recommendation for study termination will be considered by the TMG, with the CPI instigating an early termination plan.

In addition, the CPI or funder may implement early termination of the study due to administrative reasons. All participants will receive appropriate medical treatment as determined by their treating physician.

#### Adverse event reporting and harms {22}

Adverse events (AEs) will be monitored throughout the study from the time signed informed consent is obtained until 24 h administration of the last dose of the study drug. Participants with ongoing AEs will be monitored until either resolution or stabilisation is achieved, the participant is referred for continued care to another health care professional, or until a determination of the cause being unrelated to the study drug or procedure is made or the participant is lost to follow-up.

As this study involves critically or severely ill patients, it is anticipated and expected that many participants will experience events that might be considered AEs or serious adverse events (SAEs), but are expected features of critical illness requiring intensive care. Furthermore, as participants may be incapacitated for part or all of the intervention period, the identification of AEs and SAEs will largely be the responsibility of the clinical team and research teams reviewing the participant’s health status and participant records.

Screening and identification of AEs and SAEs will be based on clinical events (from daily charts and reviews) and review of laboratory and other investigations undertaken as part of routine care. There will be no testing or investigation additional to routine care undertaken for the purpose of detection of AEs or SAEs.

Seriousness, causality, severity and expectedness will be assessed by the site Principal Investigator/delegate. As this is an unblinded trial, Investigators can take group allocation into account when assessing AEs and SAEs. Treatment-emergent adverse event (TEAE) is a secondary outcome.

Adverse events will be classified by system organ class and preferred term using Medical Dictionary for Regulatory Activities (MedDRA) within REDCap. Adverse events will be classified by system organ class and preferred term using Medical Dictionary for Regulatory Activities (MedDRA, version 22 or subsequent releases).

Medical occurrences or symptoms of deterioration that are expected due to the participant’s underlying condition should be recorded in the patient’s medical notes and only be recorded as AEs in the eCRF if medically judged to have unexpectedly worsened during the study. In addition, pre-existing conditions or diseases that occur during the study (e.g. seasonal allergies, asthma or recurrent headaches) should not be considered as adverse events unless they change in frequency or severity. AEs include any occurrences that are new in onset or aggravated in severity or frequency from the baseline condition, or abnormal results of diagnostic procedures, including laboratory test abnormalities. Lack of efficacy, aggravation, or relapse of current infection are not an (S) AE in the study.

An AE is an event occurring from the time the participant (or designated proxy) signs the informed consent through to 24 h after the final dose of study medication. AES occurring before treatment are termed Non-Treatment Emergent Adverse Events (Non-TEAE), while those occurring after the start of study medication are termed treatment-emergent adverse events. From a pharmacokinetic standpoint, it is a practice to assume a drug is effectively eliminated after 5 half-lives. For ceftolozane-tazobactam, elimination is assumed at 15 h, and 5 h for meropenem. To standardise reporting operations, events occurring up to 24 h after the final dose of treatment will be defined as TEAE.

Adverse events (SAEs and suspected unexpected serious adverse reactions (SUSARs)) and/or laboratory abnormalities identified in the protocol as critical to safety evaluations should be reported within 24 h of the site becoming aware of the event to the UQCCR trial management team by telephone or email. The appropriate serious event form should be used (sites are able to use locally available SAE templates, with prior approval from the UQCCR project team). Immediate reports should be followed promptly by detailed, written follow-up reports when all information is not included in the initial report. The immediate and follow up reports should identify participants by unique code numbers assigned to study participants rather than personal identification. The investigator must also comply with all applicable ethical and regulatory requirement/s relating to the reporting of serious adverse events.

Participants experiencing adverse events will be managed according to the standard of care or the preference of the treating clinical team.

All documented adverse events will be reported in trial publication and supplementary material.

#### Frequency and plans for auditing trial conduct {23}

No formal auditing of trial conduct will be carried out by the investigating team. The study will be conducted in accordance with the principles of the International Conference on Harmonisation Tripartite Guideline for Good Clinical Practice (ICH GCP). Before the study can commence, all required approvals will be obtained and any conditions of approvals will be met.

The study will not commence at sites until a Clinical Trial Notification (CTN) or relevant Clinical Trial Authorisation (CTA) is obtained from the appropriate National Competent, with IRB/HREC and relevant local hospital governance approval. The protocol and study conduct will comply with local laws and regulations.

The Investigator is responsible for the overall conduct of the study at the site and compliance with the protocol and any protocol amendments in accordance with the principles of ICH GCP. The Investigator may delegate specified duties to an appropriately qualified, experienced member of study site staff.

Delegated tasks must be documented on a Delegation Log and signed by all those named on the list prior to undertaking applicable study-related procedures.

The Investigator must be familiar with the IMPs, protocol and the study requirements. It is the Investigator’s responsibility to ensure that all staff assisting with the study are adequately informed about the IMPs, protocol and their trial-related duties. All site study staff will be trained on study specific activities prior to trial commencement at site. This will either be conducted face to face, or webinar or via online training materials. All training will be documented.

Audits may be conducted by local site hospital audit programme or regulatory authorities.

#### Plans for communicating important protocol amendments to relevant parties (e.g. trial participants, ethical committees) {25}

The Investigators will conduct the study in compliance with the protocol provided by the Coordinating Principal Investigator. Any modifications to the protocol regarding study objectives, study design, eligibility criteria, sample sizes, or significant changes in the study that will impact study conduct, potential benefit, or safety of the study participants will initially require agreement from the TMG/DSMC. Then, the amendment will be submitted to the IRB/HREC and appropriate competent authority or approval before implementation. When necessary, the study participants will be notified of study changes and will sign an updated informed consent form reflecting such changes. In the event that a modification is needed to eliminate an immediate hazard(s) to subjects or for other inevitable medical reasons, this can be implemented prior to regulatory approval. Any deviations from the protocol must be fully documented on source documentation and will follow necessary reporting related to non-compliance and implement correct action and preventative action plans. If the investigator deviates from the protocol or makes a change to the protocol to eliminate an immediate hazard(s) to subjects, the record should be immediately submitted to the coordinating centre and the local IRB/HREC by the Investigator including the proposed documentation related to the protocol or supporting documentation.

#### Dissemination plans {31a}

The study will be reported in accordance with the Consolidated Standard of Reporting Trials (CONSORT) for non-inferiority and equivalence randomised trials [28] and the Clinical Trials Registration and Results Information Submission rule. This trial was registered at ClinicalTrials.gov/ANZCTR prior to first participant first visit and the results will be submitted to ClinicalTrials.gov. For appropriate European sites, the study will be registered prior to competent authority approval on the European Clinical Trials Database (EudraCT). The results of the study, together with other mandated information, will be uploaded to the EU Clinical Trials Register (EUCTR) within 1 year of the end of the study. In addition, the plan is to publish the trial protocol and SAP with the final results being submitted for publication in peer-reviewed scientific journals and presented at conferences. Individual participants will not be identifiable in any publication or public presentation. Individual researchers will not publish data from the trial until the main study publication has been released. The Clinical Study Report (CSR) will be submitted to the Sponsor and relevant Ethics Committees within 1 year of the end of the study. In addition, subject to the terms and conditions of funding agreement, the Coordinating Principal Investigator will provide MSD any public presentation material with the right to review and comment and include the funder’s disclosures statement on all published material. Summaries of results will also be made available to Investigators for dissemination within their clinics (where appropriate and according to their discretion). The MERINO-3 TMG will provide a lay summary of the study results to the Investigator/delegate at sites to dissemination to participants where appropriate.

The final findings will be and in different forms of presentation at national, regional, and international medical venues addressing relevant issues. Principal investigators will be eligible for authorship on future trial publications if (1) substantial contributions to the conception or design of the work; or the acquisition, analysis, or interpretation of data for the work; (2) drafting the work or revising it critically for important intellectual content; (3) final approval of the version to be published; and (4) agreement to be accountable for all aspects of the work in ensuring that questions related to the accuracy or integrity of any part of the work are appropriately investigated and resolved. No professional writers will be used in writing future manuscripts.

## Discussion

Currently, carbapenems are the preferred treatment choice for infections due to ESBL and AmpC-producing Enterobacterales. This dogma has been strengthened by the result of the MERINO trial which failed to show non-inferiority of piperacillin-tazobactam when compared to meropenem for treatment of ceftriaxone non-susceptible *E. coli* and *Klebsiella* spp. bloodstream infection [11]. Despite this trial result, debate still exists over the use of beta-lactam/beta-lactamase inhibitors as carbapenem sparing agents, especially in the setting of non-severe urinary and biliary tract infections. The PETERPEN trial is currently enrolling and again comparing piperacillin-tazobactam against meropenem in the same participant group, except they will be excluding those with septic shock (ClinicalTrials.gov NCT03671967). The PETERPEN trial is expected to complete enrolment by April 2024. Increasing use of carbapenems as a potential driver of rising rates of carbapenem-resistant organisms is concerning. There is a great need to find suitable alternatives to carbapenems to treat these infections both for participant centred and public health reasons. Ceftolozane-tazobactam has demonstrated lower MICs to these target organisms when compared to piperacillin-tazobactam, and our trial will use double the standard dosing regimen to optimise PK/PD (3 g every 8 h), which points to ceftolozane-tazobactam being a good carbapenem-sparing candidate [23]. In addition, recording the rate of colonisation or infection with carbapenem-resistant organisms and *C. difficile* infection in our trial population will aim to identify the treatment strategy with the least ‘collateral damage’ [24].

### Trial status

MSD will have manufactured the trial drug and are awaiting distribution to main sites within the six countries recruiting for the MERINO-3 trial. To date, HREC approval for protocol version 3, 17th of February 2020 has been obtained from Royal Brisbane and Women’s Hospital as part of the Australian Mutual National Acceptance Scheme. Clinical Trial Notification to the Therapeutics Goods Administration. Each participating hospital will apply for local ethical approval and Clinical Trial Approval from the appropriate National Competent Authority prior to study commencement. Remote site initiation and training is planned. Unfortunately, due to the COVID-19 pandemic, delays in site initiation and recruitment are expected. We expect to recruit our first participant in January 2022 with an estimated date of trial completion in December 2024.

## Supplementary Information

**Additional file 1.**

**Additional file 2.**

## Abbreviations

ACCI: Age-Adjusted Charlson Comorbidity Index
ADL: Activities of daily living
AE: Adverse event
AR: Adverse reaction
BID: Twice a day (Latin; *bis in die*)
BSI: Bloodstream infection
CA: Competent authority
CDI: *Clostridium difficile* infection
CI: Confidence interval
cIAI: Complicated intra-abdominal infection
CLSI: Clinical & Laboratory Standards Institute
CPE: Carbapenemase-producing Enterobacterales
CPI: Coordinating Principal Investigator
CRAB: Carbapenem-resistant *Acinetobacter Baumanii*
CrCl: Creatinine clearance
CRE: Carbapenem-eesistant Enterobacterales
CRP: C-reactive protein
CTCAE: Common terminology criteria for adverse events
CTCAE: Common Terminology Criteria for Adverse Events
CTN: Clinical Trial Notification
CTRA: Clinical Trial Research Agreement
cUTI: Complicated urinary tract infection
DMP: Data management plan
DOOR: Desirability of Outcome Ranking
DSMB: Data and Safety Monitoring Board
DSUR: Development Safety Update Report
eCRF: Electronic case report form
EDTA: Ethylenediamine tetra-acetic acid
EMA: European Medicines Agency
ESBL: Extended-spectrum beta-lactamase
EST: End of study treatment
EUC: Electrolytes, urea and creatinine
EUCAST: European Committee on Antimicrobial Susceptibility Testing
EudraCT: European Union Drug Regulating Authorities Clinical Trials
EudraVIGILANCE: European Database for Pharmacovigilance
FBC: Full blood count
FBOS: Functional Bacteraemia Outcome Score
FDA: Food and Drug Administration
GCP: Good Clinical Practice
GDPR: General Data Protection Regulations
GMP: Good Manufacturing Practice
HAP: Hospital-acquired pneumonia
HITH: Hospital In The Home
HREC: Human Research Ethics Committee
IAI: Intra-abdominal infection
IB: Investigator’s brochure
ICH: International Conference on Harmonisation
ICU: Intensive care unit
IIT: Investigator-initiated trial
IMP: Investigational Medicinal Product
INR: International Normalized Ratio
IRB: Institutional Research Board
ISF: Investigator Site File
IV: Intravenous
LOS: Length of stay
MA: Marketing aAuthorisation
MALDI TOF: Matrix-assisted laser desorption/ionisation time of flight
MAP: Mean arterial pressure
MDR: Multi-drug resistant
MDRD eGFR: Modification of Diet In Renal Disease Estimated Glomerular Filtration Rate
MedDRA: Medical Dictionary for Regulatory Activities
MIC: Minimum inhibitory concentration
MOOP: Manual of Operations
MP: Monitoring plan
MRO: Multi-resistant organism
MRSA: Methicillin-resistant *Staphylococcus aureus*
MSD: Merck, Sharpe and Dohme
OPAT: Outpatient parenteral antimicrobial therapy
PBPs: Penicillin-binding proteins
PD: Pharmacodynamics
PI: Principal Investigator
PICF: Participant Infortmation Sheet/Consent Form
PK: Pharmacokinetics
PT: Prothrombin time
QA: Quality assurance
QC: Quality control
RCT: Randomised controlled trial
REDCap: Research Electronic Data Capture
RRT: Renal replacement therapy
Rx: Treatment
SAE: Serious adverse event
SAP: Statistics analysis plan
SAP: Statistical analysis plan
SDV: Source document verification
SOFA: Sequential Organ Failure Assessment Score
SOP: Standard Operating Procedure
SUSAR: Suspected unexpected serious adverse reaction
TBL: Total bilirubin
TID: Three times a (Latin; *ter in die*)
TMF: Trial Master File
TMG: Trial Management Group
TSC: Trial Steering Committee
UAR: Unexpected adverse reaction
ULN: Upper limit of normal
UQ: University of Queensland
UQCCR: University of Queensland Centre for Clinical Research
VAP: Ventilato-associated pneumonia
VRE: Vancomycin-resistant enterococci
