# Supplementary material for: Ceftolozane-tazobactam versus meropenem for definitive treatment of bloodstream infection due to extended-spectrum beta-lactamase (ESBL) and AmpC-producing Enterobacterales (“MERINO-3”): study protocol for a multicentre, open-label randomised non-inferiority trial
Source: Trials. 2021 Apr 22;22:301. doi: 10.1186/s13063-021-05206-8 (PMC8060904; doi:10.1186/s13063-021-05206-8)
Supplement: Supplementary file 1 — Additional file 1. [file 13063_2021_5206_MOESM1_ESM.pdf]

[Institutional Logo]

Participant Information Consent Form – Participant

The MERINO-3 Trial

**Short Title**	Comparison of two antibiotics; Zerbaxa and Meropenem for treating bloodstream infections.
**Full Title**	A Multicentre, Parallel Group Open-label Randomised Controlled Non-Inferiority Phase 3 Trial, of ceftolozane-tazobactam versus meropenem for definitive treatment of bloodstream infection due to Extended-Spectrum Beta-Lactamase (ESBL) and AmpC-producing Enterobacterales
**Protocol Number**	UQCCR-DP-AS-2019-001
**Project Sponsor**	University of Queensland, Australia
**Coordinating Principal Investigator**	Professor David Paterson, Director<br>University of Queensland Centre for Clinical Research (UQCCR)<br>Building 71/918 RBWH Herston, Brisbane<br>Queensland, Australia 4029
**Associate Investigator(s)**	Dr. Adam Stewart, UQCCR<br>Dr. Patrick Harris, UQCCR
**Location**	[Institution].

Part 1- What does my participation involve?

1 Introduction

You are being invited to take part in this research project because you have a resistant Gram-negative bloodstream infection. The research project is testing a new treatment called Ceftolozane-tazobactam (ZERBAXA®) for resistant Gram-negative bloodstream infection. Zerbaxa is currently used to treat pneumonia, and complex urinary tract and abdominal infections. However, this project will study how effective it is in treating the type of blood stream infection you have.

This Participant Information Sheet/Consent Form tells you about the research project. It explains the tests, treatments and activities involved. Knowing what is involved will help you decide if you want to take part in the research.

Please read this information carefully. Please ask questions about anything that you do not understand or want to know more about. Before deciding, you might want to talk about it with a relative, friend or your doctors.

Participation in this research is voluntary. If you do not do not wish to take part, you do not have to and you will continue to receive the best possible care.

If you decide you do want to take part in the research project, you will be asked to sign the consent section. By signing it, you are telling us that you:

- Understand what you have read
- Consent to take part in the research project
- Consent to have the tests and treatments that are described
- Consent to the use of your personal and health information as described.

After you have signed, you will be given a copy of this Participant Information and Consent Form to keep for your own records.

2 What is the purpose of this research?

Gram-negative bacteria are a type of bacteria, which commonly cause infections in your blood stream. Unfortunately, these bacteria are increasing their resistance to treatment by commonly used antibiotics. Therefore, antibiotic options for this type of infection are becoming more limited. Currently, doctors faced with this problem will prescribe a “broad spectrum” antibiotic such as meropenem. Widespread use of this ‘last-line’ type of antibiotic is associated with significant alterations in the gut’s natural flora, leading to further infections and promoting antibiotic resistance.

The purpose of this study is learn whether Zerbaxa which works against resistant Gram-negative bacteria, is equally effective as meropenem in treating your condition.

Zerbaxa has been used for over 5 years and is approved worldwide for use to treat pneumonia, complex urinary tract and abdominal infections. It is currently not an approved treatment for blood stream infections and therefore considered an investigational treatment.

Although doctors have used Zerbaxa to treat blood stream infections, this is the first large study to compare it directly with currently available treatment, and if effective, it will give doctors another treatment option for blood stream infections. Advantages of using Zerbaxa may be less harm to gut flora, reducing the development of resistant bacteria, and the preservation of "last-line" antibiotic treatment options.

This is a worldwide study, taking place in 6 countries. If you choose to take part, you will be one of up to 630 participants. This research project has been designed so researchers can interpret the results in a fair and appropriate way and avoids study doctors or participants jumping to conclusions.

The results of this research will be used by the study doctor Dr Adam Stewart to obtain a Doctor of Philosophy degree.

The study team is led by Professor David Paterson from the University of Queensland. The study is being managed by the University of Queensland Clinical Research Centre and is funded by Merck Sharp & Dohme (Australia) Pty Ltd, who are the manufacturers of Zerbaxa . The study sponsor is the University of Queensland.

3 What does participation in this research involve?

You have had bloodstream infection with a resistant Gram-negative bacteria confirmed through at least one positive blood culture test.

The study is made up of three parts: screening, treatment (5 to 14 days) and a follow-up call at 30 days after commencing treatment. All your treatment will be received while you are in hospital. No further study visits are required.

A doctor or nurse who is a member of our research team will discuss the study and assess if the study is suitable for you – part of the screening phase.

If the study is suitable, and you agree to participate, the following procedures and activities will be carried out. These may be in addition to treatment or laboratory tests your doctor may be performing as part of your clinical care.

1) **Information about you:** Information about your age, previous and current illnesses, medications including known allergies will be obtained from you or from your medical record throughout the study period. In addition, the study team will collect your contact details so they can contact you at the end of the follow-up period and send you a copy of study results.

2) **Treatment Allocation**: You will be randomly assigned to either meropenem or Zerbaxa antibiotic treatment – a bit like tossing a coin, but done by a computer program. Each participant will have the same tests and measurements related to study participation so that any differences in outcome between the two groups may be attributable to the allocated treatment.

3) **Treatment:** You will receive the treatment intravenously up to 3 times a day (every 8 hours). For meropenem a maximum of 1g over 30 minutes; or 3 grams of Zerbaxa over 60 minutes. You will receive the antibiotic for a minimum of five days and up to 14 days if your treating doctor considers it necessary. Your doctor

may add a second antibiotic if intra-abdominal infection is suspected or if certain Gram-positive bacterial infections are present. This treatment will be part of your routine clinical care. If you are already receiving trimethoprim/sulfamethoxazole, your doctor may decide to continue this treatment. Your doctor may decide to switch you to orally administered antibiotics after 5 days of study drug treatment in order that you can return home, if you are well enough to do so. This is the usual standard treatment for your infection. The study team will also record some or all of your prescribed medications and any new illnesses or side-effects you may experience.

4) **Blood and laboratory tests:** These tests are routinely taken as part of your clinical care including; full blood count, kidney and liver function. The doctors and study team will use these test results to determine the appropriate dose of study antibiotic for you. In addition, the study team will record test results as part of the study.

   For women of childbearing potential, a pregnancy test may be required for study inclusion, if not already carried out by your clinical team. A positive pregnancy test will mean the study is not suitable for you.

   Your treating doctor may collect additional blood cultures as part of normal clinical care; for example if you have a fever. The study team will also record all of these results.

5) **Follow-up:** Information about how you responded to the antibiotic treatment will be obtained from you or your medical record. We are most interested in how you are going after 30 days from the start of your treatment, so you may receive a phone call from the study team at that point.

There are no additional costs associated with participating in this research project, nor will you be paid. All medications, tests and medical care required as part of the research project will be provided to you free of charge.

4 What do I have to do?

The study is designed to reflect standard clinical care, which means there are limited additional actions you need to take. As part of your care plan your treating doctor would have prescribed antibiotic treatment. The only difference by taking part in this study is that you will be randomly allocated to antibiotic treatment; either meropenem or Zerbaxa for a period of 5 to 14 days, as deemed appropriate by your treating physician. There are no additional tests (except a pregnancy test, if applicable and routine bloods on day 1 and day 5 of treatment if the treating clinicians have not already taken them as part of your routine care), procedures or hospital visits. However, at 30 days after commencing study treatment, the study team may need to call to assess your progress.

5 Other relevant information about the research project

This is an international investigator initiated project including researchers from Australia, Singapore, Lebanon, Saudi Arabia, Italy and Spain working in collaboration.

This study will recruit up to 630 participants and we expect <x> number of people will be take part in this project from <institute name>.

6 Do I have to take part in this research project?

Participation in any research project is voluntary. If you do not wish to take part, you do not have to. If you decide to take part and later change your mind, you are free to withdraw from the project at any stage.

If you do decide to take part, you will be given this Participant Information and Consent Form to sign and you will be given a copy to keep.

Your decision whether to take part or not take part, or take part and later withdraw will not affect your routine treatment, or your relationships with the treating team, or the [Institution].

7 What are the alternatives to participation?

You do not have to take part in this research project to receive treatment at this hospital. Other options are available; these include continuing on the standard of care treatment recommended by your treatment team. Your study doctor will discuss these options with you before you decide if you should take part in this research project.

8 What are the possible benefits of taking part?

All patients will be given the same high level of care that is routinely given to them by their hospital physicians. This study will not interfere with current or future treatments and care provided to the participant by the doctors. You may not personally receive a benefit from participating in the study but the information may help future patients.

9 What are the possible risks and disadvantages of taking part?

Medical treatments can cause side effects. You may have none, some or all of the effects listed in the tables below, related to the study medication and they may be mild, moderate or severe. If you have any of these side effects, or are worried about them, talk with your study doctor. Your study doctor will also be looking out for side effects.

There may be side effects that the researchers do not expect or do not know about and that may be serious. Your study doctor will be monitoring any new or unusual symptoms that you develop.

Many side effects go away shortly after treatment ends. However, sometimes side effects can be serious, long lasting or permanent. If a severe side effect or reaction occurs, your study doctor may need to stop your treatment. Your doctor will discuss the best way of managing any side effects with you.

As part of your routine care you may also receive additional medicines. These may also cause side-effects. You will be closely monitored by your treating and study doctors. As part of the study all changes to your health will be treated, recorded and reported as required.
The frequency of side effects are described as follows:

Common	Uncommon	Rare
Between 1 in 10 and 1 in 100 people are affected	Between 1 in 100 and 1 in 1,000 people are affected	Between 1 in 1,000 and 1 in 10,000 people are affected

The following two tables describes the side effects reported and how many people they have affected when taking the medication.

Ceftolozane-tazobactam (Zerbaxa)

Side Effect	Frequency Category
Rash or other reaction where the injection was given	Common
Diarrhoea, nausea, vomiting, constipation or abdominal pain	Common
Low blood pressure, fever	Common
Increase in liver enzymes (from blood tests)	Common
Blood test abnormalities such as high platelets, decreased potassium	Uncommon
Insomnia, anxiety or dizziness	Common
Infections such as thrush, urinary tract infections or infectious diarrhoea	Uncommon
Other blood test abnormalities including low red blood cells, low glucose, low magnesium and phosphate	Uncommon
Heart and brain disorders such as stroke and abnormal heart rhythms	Uncommon
Shortness of breath	Uncommon
Bloating, heartburn and abdominal cramping	Uncommon
Reduced kidney function and kidney failure	Uncommon

Meropenem

Side Effect	Frequency Category
Rash or other reaction where the injection was given	Common
Diarrhoea, nausea, vomiting or abdominal pain	Common
Blood test abnormalities such as high platelets, deranged liver function tests	Common
Headache	Common
Infections such as thrush, urinary tract infections or infectious diarrhoea	Uncommon
Other blood test abnormalities high bilirubin, low platelets, low white blood cells, low red blood cells	Uncommon
Lip, tongue and throat swelling (anaphylaxis)	Uncommon
Pins and needles	Uncommon
Reduced kidney function and kidney failure	Uncommon
Seizures	Rare

Having a drug injected or blood taken may cause discomfort, bruising, minor infection or bleeding. If this happens, it can be easily treated.

Pregnancy

The effects of Zerbaxa on the unborn child and on the newborn baby are not known. Because of this, it is important that research project participants are not pregnant or breast- feeding and do not become pregnant during the course of the research project. You must not participate in the research if you are pregnant or trying to become pregnant, or breast feeding. If you are female and child-bearing is a possibility, you will be required to undergo a pregnancy test prior to commencing the research project. In the unlikely event you become pregnant whilst participating in the research project, you should advise your study doctor immediately. Your study doctor will withdraw you from the research project and advise on further medical attention should this be necessary. You must not continue in the research if you become pregnant.

Possible Treatment Failure

Serious infections like bloodstream infections may not always be successfully treated with the initial antibiotic chosen. If your infection is not responding to the antibiotic assigned to you during this study, an alternative antibiotic may be chosen by your doctor. In some cases, other interventions may be necessary such as surgery or removal of intravenous lines. If these interventions are necessary they will be discussed with you by your doctor.

10 What will happen to my test samples?

During the study, standard of care blood cultures will be collected from you. The bacteria from these blood tests will be isolated and shipped to The University of Queensland Centre for Clinical Research (UQCCR) for future testing. During your stay in hospital, you may also have standard of care blood tests to assess you clinical response to your treatment. No blood or urine samples will be stored for study purposes.

11 What if new information arises during this research project?

Sometimes during the course of a research project, new information becomes available about the treatment that is being studied. If this happens, your study doctor will tell you about it and discuss with you whether you want to continue in the research project. If you decide to withdraw, your study doctor will make arrangements for your regular healthcare to continue. If you decide that you can continue in the research project you will be asked to sign an updated consent form.

Also, on receiving new information, your study doctor might consider it to be in your best interests to be withdrawn from the research project. If this happens, the doctor will explain the reasons and arrange for your regular healthcare to continue.

12 Can I have other treatments during this research project?

While you are participating in this research project, you may able to take some or all of the medications or treatments you have been taking for your condition or other reasons. It is important to tell the your study doctor and the study staff about any treatments or medications you may be taking, including over-the-counter medications, vitamins or herbal remedies, acupuncture or other alternative treatments. Your study doctor should also explain to you which treatments or medications need to be stopped for the time the you are involved in the research project.

13 What if I withdraw from this research project?

You may wish to stop study treatment, which you can do at any time without giving a reason, and without affecting your medical care. Your treating physician will discuss this with you, as you may require additional antibiotic treatment. Any information already collected will be retained to ensure that the results of the research project can be measured accurately and to comply with law.

If you do decide to stop the study treatment, your study team will ask if you will agree to allow them to follow your progress. This may include a telephone call 30 days after starting your study treatment. To record your decisions you will be requested to sign a withdrawal of consent form.

You should be aware that data collected up to the time you withdraw will be part of the research project results. If you do not agree to this, you must tell your study doctor before you join the research project.

14 Could this research project be stopped unexpectedly?

This research project may be stopped unexpectedly for a variety of reasons. These may include:
- Unacceptable side effects
- The antibiotic being shown not to be effective
- The antibiotic being shown to work and not need further testing
- Decisions made by local regulatory/health authorities

15 What happens when the research project ends?

You will be followed up by the study team up until 30 days after starting the treatment. The follow up may be in the form of a phone call to see how you are doing. You will have stopped the study treatment during your stay in hospital. The treating doctor may prescribe an oral antibiotic when you are discharged.

You should attend all your normal scheduled hospital and doctors' appointments.

When you join this research project, you will be invited to receive the final results of the research. If you agree, the local study team will collect and securely store your contact details to enable them to send you a summary of the project results. The anticipated end of study is 2024.

A description of this study will be available on http://www.clinicaltrials.gov. You can search this web site at any time. The study results will also be posted on the website but will not include information that can identify you.

Part 2 How is the research project being conducted?

16 What will happen to information about me?

By signing the Consent Form, you consent to the study doctor and relevant research staff collecting and using personal information about you for the research project. Any information obtained in connection with this research project that can identify you will remain confidential.

Your hospital will use your personal information for your care, to collect accurate data for the study, and if necessary, to contact you about the study.

Information about you may be obtained from your health records held at this and other health services for the purpose of this research. By signing the consent form, you agree to the study team accessing health records if they are relevant to participation in this research project. All non-identifiable data will be held on a secure server at the University of Queensland for up to 15 years as required by law.

If you withdraw your consent to take part in the study, the hospital will not collect any further information about you for the purpose of the study, unless you agree, but will keep the information already collected about you solely for the purpose of the study. To safeguard your rights, the hospital will collect and use as little personal information as possible.

There could be the potential for future third party researchers (e.g. Merck, Sharp & Dohme, who has provided funding for this study) to access the data collected from this study for the conduct of future research. The future research would be in the same general area of research – that is, the effectiveness of antibiotics. This may involve entering the participant's data into a new database. Never at any point will your personal information ever be made accessible to anyone outside of this study's research team.

Your health records and any information obtained during the research project are subject to inspection (for the purpose of verifying the procedures and the data) by the relevant authorities and authorised representatives of the sponsor, The University of Queensland the institution relevant to this Participant Information Sheet, *[Name of institution]*, or as required by law. By signing the Consent Form, you authorise release of, or access to, this confidential information to the relevant study personnel and regulatory authorities as noted above.

It is anticipated that the results of this research project will be published and or presented in a variety of forums. In any publication and/or presentation, information will be provided in such a way that you cannot be identified, except with your permission. Data will only be presented in a non-identifiable form.

Information about your participation in this research project will be documented in your health records.

In accordance with relevant Australian *and/or [Name of state/territory]* privacy and other relevant laws, you have the right to request access to your information that is collected and stored by the study team. You also have the right to request that any information with which you disagree to be corrected. Please contact the study team member named at the end of this document if you would like to access to this information.

Your study data may be transferred to countries where the law does not protect your privacy to the same extent as the law in [insert country name]. If your data is transferred outside your country, the University of Queensland will be responsible for taking appropriate steps to protect your privacy and keep your data secure.

17 Complaints and compensation

If you have a complaint about your participation, in the first instance you should talk to a researcher involved in the study. You can also make a formal complaint. You can make a complaint to a senior member of the research team or to the Complaints Officer (details provided in Section 20.)

If you suffer any injuries or complications as a result of this study, you should contact the study doctor as soon as possible, who will assist you in arranging any appropriate medical treatment.

If you suffer any health problems as a result of participating in this research study, compensation will be provided for the reasonable costs of medical treatment to the extent such costs are not covered by your medical insurance or government health schemes. Compensation will be provided in accordance with the Medicines Australia Guidelines for Compensation for Injury Resulting from Participation in an Industry-

Sponsored Clinical Trial. A copy of these guidelines can be given to you by your study team.

Other forms of compensation may also be available, although, you may need to seek legal assistance in order to obtain compensation. If you think that some form of compensation is required, we encourage you to discuss your case with the study doctor or the hospital ethics committee (details in Section 20.).

18 Who is organising and funding the research?

This research project is being conducted by the University of Queensland (UQ). Merck, Sharp & Dohme (Australia) Pty Ltd (MSD) have provided funding for the conduct of the study. MSD may benefit financially from this research project if, for example, the project assists MSD to obtain approval for Zerbaxa to treat bloodstream infection. By taking part in this research project you agree that data generated from samples of your blood or urine, which is provided to UQ may directly or indirectly benefit from knowledge acquired through analysis of your samples. In addition, if knowledge acquired through this research leads to discoveries that are of commercial value to UQ or MSD, the study doctors or their institutions, there will be no financial benefit to you or your family from these discoveries. No member of the research team will receive a personal financial benefit from your involvement in this research project (other than ordinary wages).

19 Who has reviewed the research project?

All research in Australia involving humans is reviewed by an independent group of people called a Human Research Ethics Committee (HREC). They review research proposals to ensure that they meet ethical standards and guidelines.

The ethical aspects of this research project have been approved by the HREC of <Royal Brisbane and Women's Hospital>.

Australia Only - This project will be carried out according to The International Council for Harmonisation) Good Clinical Practice (ICH-GCP) principals and the *National Statement on Ethical Conduct in Human Research (2018)*. This statement has been developed to protect the interests of people who agree to participate in human research studies.

External to Australia – This project will be carried out according to The International Council for Harmonisation Good Clinical Practice principals (ICH-GCP) < add additional regional guidelines/conditions, if relevant> to protect the interests of people who agree to participate in human research studies

20 Further information and who to contact

The person you may need to contact will depend on the nature of your query.

If you want any further information concerning this project or if you have any medical problems which may be related to your involvement in the project (e.g. any side effects), you can contact the principal study doctor on *[phone number]* or any of the following people:

Clinical Contact Person

Name	*[Site PI Name]*
Position	*[Position]*
Telephone	*[Phone number]*
Email	*[Email address]*

For matters relating to research at the site at which you are participating, the details of the local site complaints person are:

Complaints Contact Person

Name	*[Site Governance Officer]*
Position	*[Position]*
Telephone	*[Phone number]*
Email	*[Email address]*

If you have any complaints about any aspect of the project, the way it is being conducted or any questions about being a research participant in general, then you may contact:

Reviewing HREC approving this research and HREC Executive Officer details

Reviewing HREC name	
HREC Executive Officer	
Telephone	
Email	

[Institutional Logo]

Consent Form

The MERINO-3 Trial

**Short Title**	Comparison of two antibiotics; Zerbaxa and Meropenem for treating bloodstream infections.
**Full Title**	A Multicentre, Parallel Group Open-label Randomised Controlled Non-Inferiority Phase 3 Trial, of ceftolozane-tazobactam versus meropenem for definitive treatment of bloodstream infection due to Extended-Spectrum Beta-Lactamase (ESBL) and AmpC-producing Enterobacterales
**Protocol Number**	UQCCR-DP-AS-2019-001
**Project Sponsor**	University of Queensland, Australia
**Coordinating Principal Investigator**	Professor David Paterson, Director<br>University of Queensland Centre for Clinical Research (UQCCR)<br>Building 71/918 RBWH Herston, Brisbane<br>Queensland, Australia 4029
**Associate Investigator(s)**	Dr. Adam Stewart, UQCCR<br>Dr. Patrick Harris, UQCCR
**Location**	[Institution].

<u>Declaration by Participant</u>

1. I have read the Participant Information Sheet or someone has read it to me in a language that I understand. I have had the opportunity to consider the information, ask questions and have had these answered satisfactorily.

2. I understand the purposes, procedures and risks of the research described in the project.

3. I give permission for my doctors, other health professionals, hospitals or laboratories outside this hospital to release information concerning my conditions and treatment which is needed for this research and understand that such information will remain confidential.

4. I understand that relevant sections of my medical notes and data collected during the study, may be looked at by individuals from regulatory authorities and/or from representatives of the study's sponsor, The University of Queensland, where it is relevant to my taking part in this research. I give permission for these individuals to have access to my records.

5. I freely agree to participate in this research project as described and understand that I am free to withdraw at any time during the study without affecting my future health

care. I understand that all information collected prior to withdrawal will be used solely for the purpose of this study.

6. I consent to the storage and use of non-identifiable data in the relevant section of the Participant Information Sheet, for:

- This specific research project
- Other research that is closely related to this research project.

7. I understand that I will be given a signed copy of this document to keep.

8.

Name of Participant (please print) ______________________________

Signature ______________________ Date ______________________

Name of Witness* to Participant's Signature (please print) ______________________________

Signature ______________________ Date ______________________

* Witness is not to be the investigator, a member of the study team or their delegate. In the event that an interpreter is used, the interpreter may not act as a witness to the consent process. Witness must be 18 years or older.

Declaration by Study Doctor/Senior Researcher†

I have given a verbal explanation of the research project, its procedures and risks and I believe that the participant has understood that explanation.

Name of Study Doctor/ Senior Researcher† (please print) ______________________________

Signature ______________________ Date ______________________

† A senior member of the research team must provide the explanation of, and information concerning, the research project.

Note: All parties signing the consent section must date their own signature.

[Institutional Logo]

Form for Withdrawal of Participation

The MERINO-3 Trial

**Short Title**	Comparison of two antibiotics; Zerbaxa and Meropenem for treating bloodstream infections.
**Full Title**	A Multicentre, Parallel Group Open-label Randomised Controlled Non-Inferiority Phase 3 Trial, of ceftolozane-tazobactam versus meropenem for definitive treatment of bloodstream infection due to Extended-Spectrum Beta-Lactamase (ESBL) and AmpC-producing Enterobacterales
**Protocol Number**	UQCCR-DP-AS-2019-001
**Project Sponsor**	University of Queensland, Australia
**Coordinating Principal Investigator**	Professor David Paterson, Director<br>University of Queensland Centre for Clinical Research (UQCCR)<br>Building 71/918 RBWH Herston, Brisbane<br>Queensland, Australia 4029
**Associate Investigator(s)**	Dr. Adam Stewart, UQCCR<br>Dr. Patrick Harris, UQCCR
**Location**	[Institution].

<u>Declaration by Participant</u>

I wish to withdraw from participation in the above research project and understand that such withdrawal will not affect my routine treatment, my relationship with those treating me or my relationship with *[Institution]*.

I consent to the following as described in the Participant Information Sheet:
(please tick, and initial choice):

- ☐ Withdraw from treatment and agree to further information about my progress be collected for the purposes of this study
- ☐ Withdraw from treatment and **DO NOT** agree to any further information to be collected for the purposes of this study

Name of Participant (please ____

Signature __________________________Date __________________________

In the event that the participant's decision to withdraw is communicated verbally, the Study Doctor/Senior Researcher will need to provide a description of the circumstances below.

Declaration by Study Doctor/Senior Researcher†

I have given a verbal explanation of the implications of withdrawal from the research project and I believe that the participant has understood that explanation.

Name of Study Doctor/ Senior Researcher† (please print)	______________________________
Signature ______________________	Date ______________________

† A senior member of the research team must provide the explanation of and information concerning withdrawal from the research project.

Note: All parties signing the consent section must date their own signature.
