# Supplementary material for: Ceftolozane-tazobactam versus meropenem for definitive treatment of bloodstream infection due to extended-spectrum beta-lactamase (ESBL) and AmpC-producing Enterobacterales (“MERINO-3”): study protocol for a multicentre, open-label randomised non-inferiority trial
Source: Trials. 2021 Apr 22;22:301. doi: 10.1186/s13063-021-05206-8 (PMC8060904; doi:10.1186/s13063-021-05206-8)
Supplement: Supplementary file 2 — Additional file 2. [file 13063_2021_5206_MOESM2_ESM.docx]

WHO Trial Registration Data Set (Version 1.3.1)

| 1. Primary Registry and Trial Identifying Number | ClinicalTrials.gov identifier: NCT04238390 |
| --- | --- |
| 1. Date of Registration in Primary Registry | January 23, 2020 |
| 1. Secondary Identifying Numbers | Sponsor Issues Trial Number: UQCCR-DP-AS-2019-001 |
| 1. Source(s) of Monetary or Material Support | Merck Sharp & Dohme (Australia) Pty Limited investigator Initiated Funding |
| 1. Primary Sponsor | University of Queensland, St Lucia, Queensland, Australia, 4072 |
| 1. Secondary Sponsor | N/A |
| 1. Contact for Public Queries | Professor David Paterson, University of Queensland Centre for Clinical Research, Building 71/918 RBWH Herston, Brisbane City, QLD 4029  +61 7 33466074 |
| 1. Contact for Scientific Queries | Professor David Paterson, University of Queensland Centre for Clinical Research, Building 71/918 RBWH Herston, Brisbane City, QLD 4029  +61 7 33466074 |
| 1. Public Title | MERINO 3 |
| 1. Scientific Title | Ceftolozane-tazobactam versus meropenem for definitive treatment of bloodstream infection due to Extended-Spectrum Beta-Lactamase (ESBL) and AmpC-producing Enterobacterales (“MERINO-3”): study protocol for a multicentre, open-label randomised non-inferiority trial |
| 1. Countries of Recruitment | Australia, Singapore, Lebanon, Saudi Arabia, Spain, Italy |
| 1. Health Condition(s) or Problem(s) Studied | Bloodstream infection due to Extended-Spectrum Beta-Lactamase (ESBL) and AmpC-producing Enterobacterales |
| 1. Intervention(s) | Meropenem 1 gram or ceftolozane-tazobactam 3 grams, will both be administered every 8 hours intravenously as per product information guidelines. Meropenem will be administered over 30 minutes and ceftolozane-tazobactam over 60 minutes |
| 1. Key Inclusion and Exclusion Criteria | Inclusion criteria:  1. Qualifying bloodstream infection is defined as at least one peripheral blood culture draw demonstrating Enterobacterales with proven non-susceptibility to third generation cephalosporins or a cephalosporin susceptible chromosomal AmpC-producing Enterobacterales (*Enterobacter* spp., *Klebsiella aerogenes*, *Citrobacter freundii*, *Morganella morganii*, *Providencia* spp. or *Serratia marcescens*). Non-susceptibility to third generation cephalosporins (any one of ceftriaxone, cefotaxime or ceftazidime) is defined by susceptibility breakpoints used in the local testing laboratory (either EUCAST or CLSI). Bacterial identification to species level will be performed using standard laboratory methods (e.g. MALDI-TOF) and susceptibility testing (e.g. VITEK2) according to local practice and standards. This may include newly validated methods such as the Accelerate Pheno^TM^ system.  2. Participant is aged 18 years and over (21 and over in Singapore)  3. The participant or approved proxy is able to provide informed consent  4. ≤72 hours has elapsed since the first positive qualifying (index) blood culture collection  5. Expected to receive IV therapy for ≥5 days.  Exclusion criteria:  1. Known hypersensitivity to a cephalosporin or a carbapenem, or anaphylaxis to beta-lactam antibiotics.  2. Participant with significant polymicrobial bloodstream infection (i.e. not a contaminant)  3. Treatment is not with the intent to cure the infection (i.e. palliative intent) or the expected survival is ≤5 days  4. Participant is pregnant or breast-feeding  5. Use of concomitant antimicrobials with known activity against Gram-negative bacilli (except trimethoprim/sulfamethoxazole for Pneumocystis prophylaxis and when adding metronidazole for suspected IAI) in the first 5 days post-randomisation  6. Participants with creatinine clearance (CrCl) <15 mL/minute (determined by Cockcroft-Gault formula or Modification of Diet in Renal Disease (MDRD) formula) or on renal replacement therapy  7. Previously randomised in the MERINO-3 trial or concurrently enrolled in another therapeutic antibiotic clinical trial  8. Blood culture isolate with in-vitro resistance to either meropenem or ceftolozane-tazobactam (known either at time of enrolment or during the course of study treatment, in which case the participant will be withdrawn). |
| 1. Study Type | Phase 3 open-label, randomised, parallel group non-inferiority trial |
| 1. Date of First Enrollment | January 2022 |
| 1. Sample Size | Planning to enrol 600 participants |
| 1. Recruitment Status | Pending: participants are not yet being recruited |
| 1. Primary Outcome(s) | 30 day all-cause mortality post-randomisation |
| 1. Key Secondary Outcome(s) | 1. 14 day all-cause mortality post-randomisation  2. Clinical and microbiologic success at day 5 post-randomisation (participant alive, fever resolved <38°C, SOFA score or modified SOFA score improved, absence of growth of index organism from blood culture)  3. Functional Bacteraemia Score (FBS) at 30 days post-randomisation  4. Rates of microbiological relapse (growth of same organism as index blood culture up to 30 days post-randomisation)  5. Growth of a new organism from blood cultures (not a contaminant) up to and including 30 days post-randomisation  6. Length of hospital and ICU stay with each regimen  7. Treatment emergent serious adverse events  8. Rates of Clostridium Difficile Infection (CDI) post treatment  9. Rates of colonization and/or infection with multi-resistant bacterial organisms (MROs)  10. Desirability of Outcome Ranking (DOOR) with partial credit for both groups. |
| 1. Ethics Review | Approved 17^th^ February, 2020  Royal Brisbane & Women’s Hospital Human Research Ethics Committee  RBWH-Ethics@health.qld.gov.au |
| 1. Completion Date | January 2024 |
| 1. Summary Results | N/A |
| 1. IPD Sharing Statement | IPD will be shared to contribute to future meta-analyses |
